# Gender differences and defensive coping behavior in patients with inflammatory bowel disorders

**DOI:** 10.1192/j.eurpsy.2021.1613

**Published:** 2021-08-13

**Authors:** N. Chernus, R. Gorenkov, S. .Sivkov, S. Sivkova, A. Sivkov, T. Savina

**Affiliations:** 1 Department Of Outpatient Therapy, I.M. Sechenov First Moscow State Medical University: Moscow, Russia, Moscow, Russian Federation; 2 Scientific Substantiation Of Health Preservation Of The Population Of The Russian Federation, The N. A. Semashko National Research Institute of Public Health, Moscow, Russian Federation; 3 The Department Of Clinical Pharmacology And Internal Diseases Propaedeutics, the I.M. Sechenov First Moscow State Medical University, Moscow, Russian Federation

**Keywords:** gender, psychological defense mechanisms, coping behaviors, maladaptation

## Abstract

**Introduction:**

Inflammatory bowel disorders (IBD) are chronic diseases with severe course. In this regard, research aimed at identifying adaptive behavior styles potentially significant for individual resilience to disease-related stress is of particular importance

**Objectives:**

The study population included 45 patients with the inflammatory bowel disorders: 19 male, 26 female (mean age 36,0±4,8), whose clinical and experimental psychological characteristics were studied.

**Methods:**

The following methods were used: ‘Life Style Index’ by R. Plutchik, Н. Kellerman, ‘Ways of Coping’ by R. Lazarus, S. Folkman.

**Results:**

The experimental psychological study revealed interdependence of psychological defense mechanisms and coping behaviors. Thus, in female patients, such psychological defense mechanisms as ‘denial r=-0,51’ and ‘compensation г=-0,43’ showed negative correlation with ‘planning problem-solving’ coping strategy and positive correlation with such coping strategies, as ‘escape - avoidance г=0,38’ and ‘confrontation г=0,32’ р<0,05; in male patients, such psychological defense mechanisms as ‘regression г= -0,41;’ and ‘displacement г= -0,30’ demonstrated negative correlation with ‘planning problem-solving’ and ‘exercising self-control’, but positive correlation with such coping strategies, as ‘escape - avoidance г= 0,34’, ‘confrontation г= 0,40;’, р<0,05. Maladaptive attitude towards disease correlated with avoidance reactions in both male and female patients, which is indicated by the central rank position in disease coping structure of ‘confrontation’ coping strategy М=69,3±0,1, along with insufficient utilization of ‘planning problem-solving’ coping strategy М=39.3± 0,1, р< 0,001.

**Conclusions:**

The identified manifestations of psychological maladaptation in both male and female inflammatory bowel disorder patients are moderately pronounced, but require psychotherapeutic correction

